# IRE1 Endoribonuclease Activity Modulates Hypoxic HIF-1α Signaling in Human Endothelial Cells

**DOI:** 10.3390/biom10060895

**Published:** 2020-06-11

**Authors:** Adrianna Moszyńska, James F. Collawn, Rafal Bartoszewski

**Affiliations:** 1 Department of Biology and Pharmaceutical Botany, Medical University of Gdansk, 80210 Gdansk, Poland; 2 Department of Cell, Developmental and Integrative Biology, University of Alabama at Birmingham, Birmingham, AL 35294, USA; jcollawn@uab.edu

**Keywords:** RIDD, HIF1A, XBP1, ERN1, hypoxia

## Abstract

While the role of hypoxia and the induction of the hypoxia inducible factors (HIFs) and the unfolded protein response (UPR) pathways in the cancer microenvironment are well characterized, their roles and relationship in normal human endothelium are less clear. Here, we examined the effects of IRE1 on HIF-1α protein levels during hypoxia in primary human umbilical vein endothelial cells (HUVECs). The results demonstrated that HIF-1α levels peaked at 6 h of hypoxia along with two of their target genes, *GLUT1* and *VEGFA*, whereas at up to 12 h of hypoxia the mRNA levels of markers of the UPR, *IRE1*, *XBP1s*, *BiP*, and *CHOP*, did not increase, suggesting that the UPR was not activated. Interestingly, the siRNA knockdown of IRE1 or inhibition of *IRE1* endonuclease activity with 4µ8C during hypoxia significantly reduced HIF-1α protein without affecting *HIF1A* mRNA expression. The inhibition of the endonuclease activity with 4µ8C in two other primary endothelial cells during hypoxia, human cardiac microvascular endothelial cells and human aortic endothelial cells showed the same reduction in the HIF-1α protein. Surprisingly, the siRNA knockdown of *XBP1s* during hypoxia did not decrease the HIF1α protein levels, indicating that the IRE1-mediated effect on stabilizing the HIF1α protein levels was XBP1s-independent. The studies presented here, therefore, provide evidence that IRE1 activity during hypoxia increases the protein levels of HIF1α in an XBP1s-independent manner.

## 1. Introduction

In order to adjust to stress conditions, cells undergo critical adaptive responses during hypoxia. This includes the up-regulation of hypoxia-inducible factors (HIFs) and potentially the induction of the unfolded protein response (UPR). The UPR pathway consists of distinct signaling axes that are mediated by three endoplasmic reticulum (ER) transmembrane stress sensors: activating transcription factor 6 (ATF6), protein kinase RNA-like endoplasmic reticulum kinase (PERK) and Inositol-requiring transmembrane kinase/endoribonuclease 1α (IRE1α) [1,2,3]. The activation of this pathway protects the cell against protein misfolding during hypoxia [4,5,6,7,8,9,10,11]. Despite the importance of both HIFs and the UPR pathway for the cancer microenvironment and cardiovascular disorders, the relationship between the HIFs and the UPR is poorly understood, especially in the normal human endothelium [12]. Although the HIF-dependent activation of the PERK axes has been reported in both cancer and normal cells [4,13], the hypoxic activation of ATF6 and IRE1 signaling remains ambiguous. Notably, the UPR during unmitigated stress directs cells toward apoptosis, whereas the IRE1 axes serve as a molecular timer for the cell fate decision process. The activation of IRE1α leads to the reduction of protein synthesis through regulated IRE1-dependent decay (RIDD), which results in the degradation of selected mRNAs [14]. Additionally, the active spliced isoform of the X-box binding-protein transcription factor (XBP1s) is formed by the endoribonuclease activity of IRE1α [15], facilitating cell survival and increasing the ER’s folding capacity [15,16,17]. Furthermore, the inflammatory response and activation of autophagy and apoptosis processes led by Janus N-terminal kinase (JNK) is achieved by IRE1α kinase activity [14,18]. Although it is feasible that IRE1 signaling could also be important during hypoxia for cell survival, the activation of this pathway and its relation to HIF signaling is virtually unknown. To date, both hypoxic induction and the impairment of XBP1s have been reported in cancer cell lines [19,20,21,22,23,24,25,26,27,28], whereas IRE1-related activity that did not result in XBP1s accumulation was observed in human endothelium [29].

In this study, we have focused on the consequences of IRE1 activation on HIF-1α levels during hypoxia in human primary endothelial cells. Our findings reveal that the impairment of IRE1 activity results in reduced HIF-1α protein levels that are independent of XBP1s. Our results suggest that regulated IRE1-dependent decay of mRNA (RIDD) is an important regulator of HIF-1α protein expression during hypoxia.

## 2. Materials and Methods

### 2.1. Cell Culture

Primary human umbilical vein endothelial cells (HUVECs) (#ZHC-2301) were obtained from Cellworks and cultured in EGM-2 Endothelial Cell Growth Medium-2 BulletKit (Lonza, Visp, Switzerland). Primary human aortic ECs (HAECs) were purchased from Lonza and cultured in EGM-2 medium. Primary human cardiac microvascular ECs (HMVECs-C) were also purchased from Lonza and cultured in EGM-2MV medium. All experiments were conducted at passage 4 at a confluence of 80%. Cells were cultured in a humidified incubator (Thermo Scientific, Waltham, MA, USA) at 37 °C in 5% CO_2_ in T75 culture flasks (Falcon) before plating them into smaller culture dishes (35 or 60 mm) for RNA or protein isolation, respectively.

### 2.2. Hypoxia Induction

Hypoxia was induced in a physiological cell culture workstation InvivO_2_ (Baker Ruskin, FL, USA) designed for hypoxia research. Both the media and workstation were pre-equilibrated for 2 h prior to the experiments. Cells were maintained at 0.9% O_2_ for the time periods specified (PO_2_ was 10–12 mm Hg) [30,31]. At the same time, control cells were maintained in normoxia inside a CO_2_/O_2_ incubator (Thermo Scientific).

### 2.3. IRE1α Inhibition

Cells were treated for 6 h in normoxia or hypoxia with 20 µM final concentration of 4µ8C (Sigma-Aldrich) dissolved in DMSO (Sigma-Aldrich, St. Louis, MI, USA).

### 2.4. siRNA Transfection

HUVECs were transfected using Lipofectamine RNAiMAX (Thermo Scientific) according to the manufacturer’s protocol. All siRNAs (Ambion, Austin, TX, USA) were used at a final concentration of 40 nM: *XBP1* (ID s14915), *ERN1* (ID s200432), and Negative Control No. 1 (#4390843). After 24 h, the transfected cells were put into a hypoxia chamber for 6 h, whereas the control cells remained in an incubator with normoxic conditions.

### 2.5. RNA Isolation

Total RNA (containing both mRNA and microRNA) was isolated using a miRNeasy Mini Kit (Qiagen, Hilden, Germany). RNA concentrations were calculated based on the absorbance at 260 nm. RNA samples were stored at −70 °C until use.

### 2.6. Real Time PCR (qRT-PCR)

The TaqMan RNA-to-Ct 1-Step Kit (Thermo Scientific) was used following the manufacturer’s protocol. The relative mRNA expression levels were calculated using the 2^-ΔΔCt^ method [32] with the *18S* and *RPLP0* genes as the reference genes [33]. The TaqMan Assay IDs were: *18S* (Hs99999901_s1); *DDIT3* [alias CHOP] (Hs00358796_m1); *ERN1* [IRE1α gene] (Hs00176385_m1); *HIF1A* (Hs00153153_m1); *HSPA5* [alias BiP] (Hs00607129_gH); *RPLP0* (Hs00420895_gH); *SLC2A1* [GLUT1 gene] (Hs00892681_m1); *VEGFA* (Hs00900055_m1); *XBP1* (Hs00231936_m1); and *XBP1s* (Hs03929085_g1).

### 2.7. Western Blot Analyses

Western Blot analysis was performed as previously described [34]. Following the normalization of protein concentrations, the lysates were mixed with an equal volume of 6X Laemmli sample buffer (12% SDS, 60% glycerol, 0.06% bromophenol blue, 375 mM Tris-HCl pH = 6.8) and incubated for 5 min at 95 °C prior to separation by SDS-PAGE on a 4–15% Criterion TGX Stain-Free Gel (Bio-Rad, Hercules, CA, USA). Following SDS-PAGE, the proteins were transferred to polyvinylidene fluoride membranes (Bio-Rad) using the wet electroblotting method (300 mA, 4 °C, 90 min for one gel and 180 min for two gels). The membranes were blocked with BSA dissolved in TBS/Tween-20 (3% BSA, 0.5% Tween-20 for 1 h), followed by immunoblotting with the primary antibodies (overnight, 4 °C): mouse anti–HIF-1α (1:2000, ab16066; Abcam) and rabbit anti–β-actin (1:1000, ab1801; Abcam). After the washing steps, the membranes were incubated with goat anti-rabbit IgG (heavy and light chains) or with goat anti-mouse IgG (heavy and light chains) horseradish peroxidase-conjugated secondary antibodies (Bio-Rad) for 1 h at room temperature and detected using SuperSignal West Pico ECL (Thermo Scientific). Densitometry was performed using the Image Lab software v.4.1 (Bio-Rad).

### 2.8. Statistical Analysis

Results were expressed as means ± standard error (SEM). Statistical significance was determined using the Student’s t test (one-tailed, homoscedastic), with *p* < 0.05 considered significant.

## 3. Results

**Figure 1:**
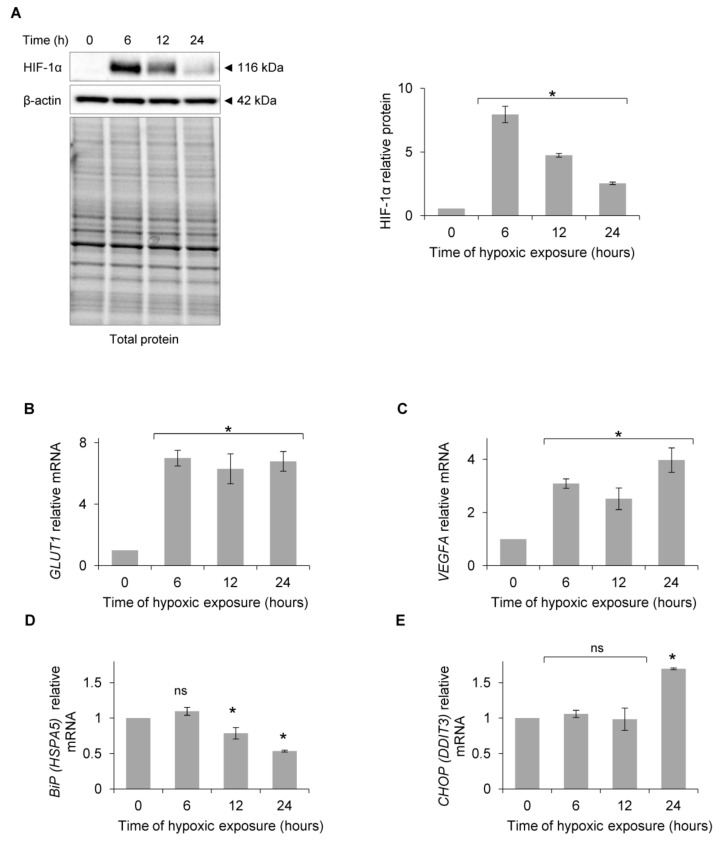
Primary HUVECs were exposed to hypoxia (0.9% O_2_) for up to 24 h. (**A**) HIF-1α protein levels were evaluated at the specified time points by Western Blotting, normalized to β-actin and total protein levels and related to the time of hypoxic exposure. The densitometry analysis is representing two independent experiments (* *p* < 0.05 was considered significant). (**B**) *GLUT1* (*SLC2A1)*, (**C**) *VEGFA*, (**D**) *BiP* (*HSPA5)* and (**E**) *CHOP* (*DDIT3*) mRNA levels were quantified by quantitative real-time PCR and normalized to *18S* and *RPLP0* rRNA levels and expressed as a fold change over normoxic samples. Data represent the mean ± SEM of four independent experiments.

To determine when the exposure of human endothelial cells to acute hypoxia results in UPR IRE1 pathway activation, we performed a time-course study and monitored the classic UPR proadaptive and apoptotic mRNA markers in primary human endothelial cells. Primary HUVECs (pooled from 10 independent donors) were exposed to hypoxia (0.9% O_2_) for up to 24 h, and HIF-1α protein levels were measured at the specified time points. As shown in Figure 1A, HIF-1α levels peaked at 6 h, and although they were reduced at 12 h and 24 h, they remained elevated during the entire 24 h time course compared to the normoxic control. The hypoxic accumulation of HIF-1α was also indicated by HIF-1 activity that resulted in the induction of mRNA for two of its transcriptional targets, the glucose transporter protein type 1 (*GLUT1* (*SLCA2A1*)*)* mRNA and vascular endothelial growth factor A (*VEGFA*) mRNA (Figure 1B,C). These results that confirm the hypoxic activation of HIF-1 signaling in HUVECs are in good agreement with previous studies including our own [34,35,36,37,38,39,40]. Surprisingly, the luminal endoplasmic reticulum protein BiP (*HSPA5)* mRNA levels, a UPR pro-adaptive activation marker [41,42,43,44], were reduced after 12 h of exposure to hypoxia (Figure 1D), while the mRNA levels of apoptotic C/EBP homologous protein (*CHOP* (*DDIT3*)) [41,43,44,45] were elevated only after 24 h of exposure to hypoxia (Figure 1E). Thus, the exposure of HUVECs to hypoxia did not result in ER stress and the subsequent activation of UPR signaling during the earlier time points of up to 12 h.

**Figure 2:**
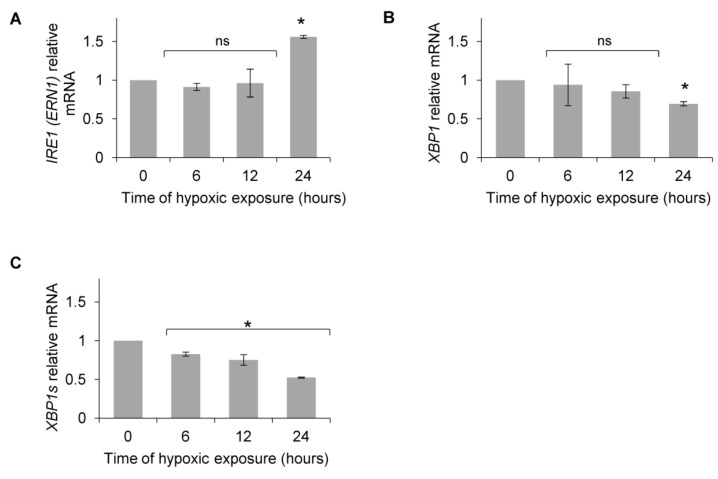
Primary HUVECs were exposed to hypoxia (0.9% O_2_) for up to 24 h. (**A**) *IRE1* *(ERN1)*, (**B**) *XBP1* (total) and (**C**) *XBP1s* (spliced) mRNA levels were quantified by quantitative real-time PCR and normalized to 18S and *RPLP0* rRNA levels and expressed as a fold change over normoxic samples. Data represent the mean ± SEM of four independent experiments (* *p* < 0.05 was considered significant).

Based on previous reports that postulated that XBP1s, a product of the UPR IRE1 activation pathway, could potentiate the HIF-1-dependent induction of *VEGF* mRNA [46,47,48,49], we followed *IRE1* (*ERN1*) and *XBP1* mRNA levels. As shown in Figure 2A, *IRE1* mRNA levels did not increase during the first 12 h, and total *XBP1* mRNA only went down after 24 h (Figure 2B), whereas *XBP1s* was significantly downregulated after 6 h and throughout the rest of the time course (Figure 2C).

**Figure 3:**
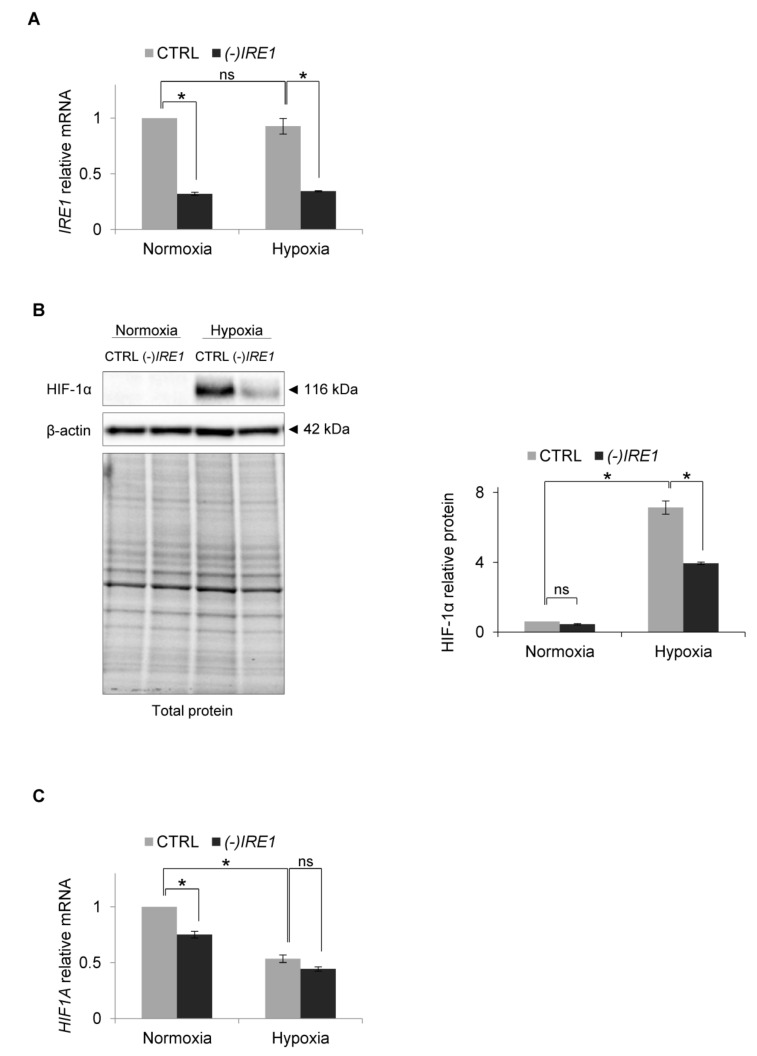
*IRE1* knockdown affects HIF1α protein level in hypoxia in HUVECs. (**A**) *IRE1* mRNA levels after *IRE1* knockdown. (**B**) HIF-1α protein levels after *IRE1* knockdown in normoxia and hypoxia were evaluated by Western Blotting, normalized to β-actin and total protein levels and related to hypoxia (CTRL). The densitometry analysis is representing two independent experiments (* *p* < 0.05 was considered significant). (**C**) *HIF1A* mRNA levels after *IRE1* knockdown. (**A**) and (**C**) mRNA levels were quantified by quantitative real-time PCR, normalized to 18S and RPLP0 rRNA levels and expressed as fold changes over normoxic samples. Data represent the mean ± SEM of two independent experiments.

Since this result suggested that hypoxia may prevent XBP1s signaling, we assessed the consequences of inactivation of the IRE1 pathway on HIF-1α accumulation during hypoxia. Using siRNA inhibition, we impaired *IRE1* mRNA expression by about 50% (Figure 3A) and followed HIF-1α protein and mRNA levels in HUVECs cultured in normoxia and exposed to hypoxia for 6 h, a time at which there was a maximal accumulation of HIF-1α protein. Interestingly, *IRE1* silencing resulted in a dramatic reduction of HIF-1α protein accumulation in hypoxia (Figure 3B), while the hypoxic *HIF1A* mRNA levels remained relatively unaffected (Figure 3C). This suggested that IRE1 was somehow contributing to the accumulation of HIF-1α during hypoxia.

**Figure 4:**
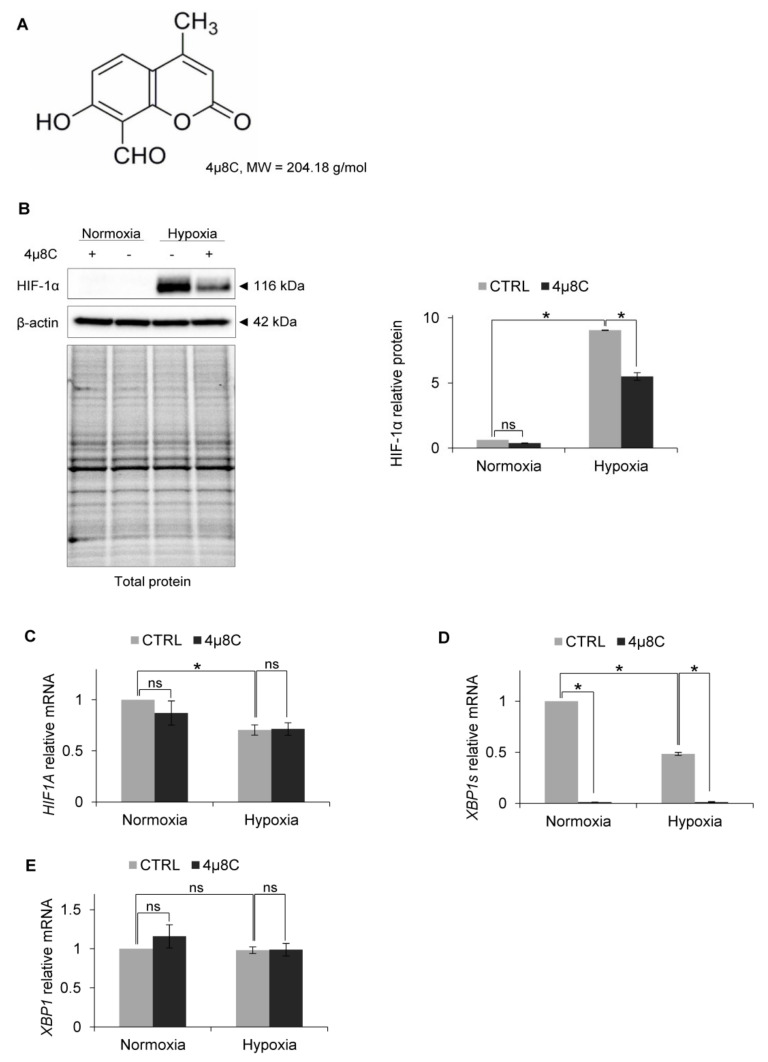
IRE1α inhibition by 4µ8C in hypoxia results in HIF-1α protein reduction in HUVECs. (**A**) Structural formula of 4µ8C. (**B**) HIF-1α protein levels after IRE1α inhibition by 4µ8C in normoxia and hypoxia were evaluated by Western Blotting, normalized to β-actin and total protein levels and related to hypoxia (CTRL). The densitometry analysis is representing two independent experiments (* *p* < 0.05 was considered significant). (**C**) *HIF1A*, (**D**) *XBP1s* (spliced) and (**E**) *XBP1* (total) mRNA levels were quantified by quantitative real-time PCR, normalized to *18S* and *RPLP0* rRNA levels and expressed as a fold change over normoxic samples. Data represent the mean ± SEM of two independent experiments.

To test the idea that IRE1 RIDD activity mediated this effect, we used a specific inhibitor of RIDD activity: 4µ8C [50] (Figure 4A). As shown in Figure 4B, in the HUVECs that were treated with 4µ8C and exposed to hypoxia, HIF-1α protein levels were reduced in a similar manner as in the cells with *IRE1* knockdown, while the *HIF1A* mRNA levels remained unchanged (Figure 4C). Notably, the 4µ8C was effectively inhibiting IRE1 endoribonuclease activity, both in normoxia and hypoxia, and thus significantly reducing *XBP1s* mRNA levels (Figure 4D), without affecting total XBP1 expression (Figure 4E). This suggested that IRE1 was active in both normoxia and hypoxia. Furthermore, despite *XBP1s* mRNA reduction during hypoxia, this transcription factor may still be crucial for hypoxic HIF-1α stabilization.

**Figure 5:**
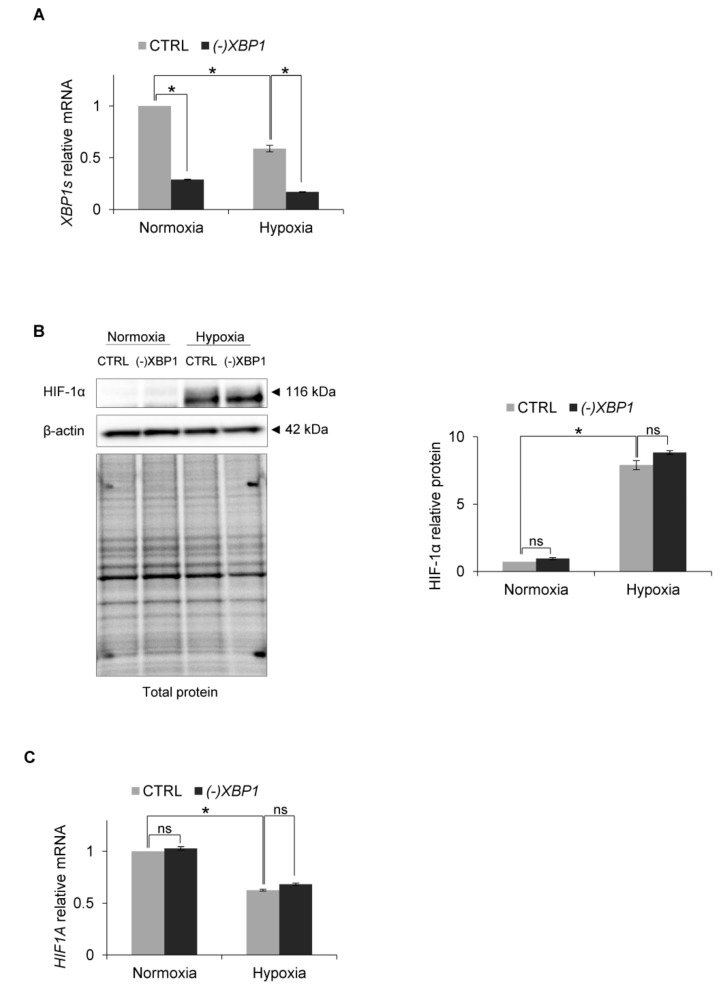
*XBP1* knockdown in hypoxia in HUVECs. (**A**) *XBP1s* (spliced) mRNA levels after *XBP1* knockdown. (**B**) HIF-1α protein levels after *XBP1* knockdown in normoxia and hypoxia were evaluated by Western Blotting, normalized to β-actin and total protein levels and related to hypoxia (CTRL). The densitometry analysis is representing two independent experiments (* *p* < 0.05 was considered significant). (**C**) *HIF1A* mRNA levels after *XBP1* knockdown. The mRNA levels were quantified by quantitative real-time PCR and normalized to *18S* and *RPLP0* rRNA levels and expressed as a fold change over normoxic samples. Data represent the mean ± SEM of two independent experiments (* *p* < 0.05 was considered significant).

Therefore, to test this, we efficiently knocked down *XBP1s* expression with specific siRNA, during both normoxic and hypoxic conditions (Figure 5A) and followed related HIF-1α protein and mRNA levels. As shown in Figure 5B,C, *XBP1s* silencing had no effect on HIF-1α protein or mRNA levels respectively. Therefore, although IRE1-RIDD activity leads to the accumulation of HIF-1α protein in human endothelial cells exposed to hypoxia, this process is XBP1s-independent.

**Figure 6:**
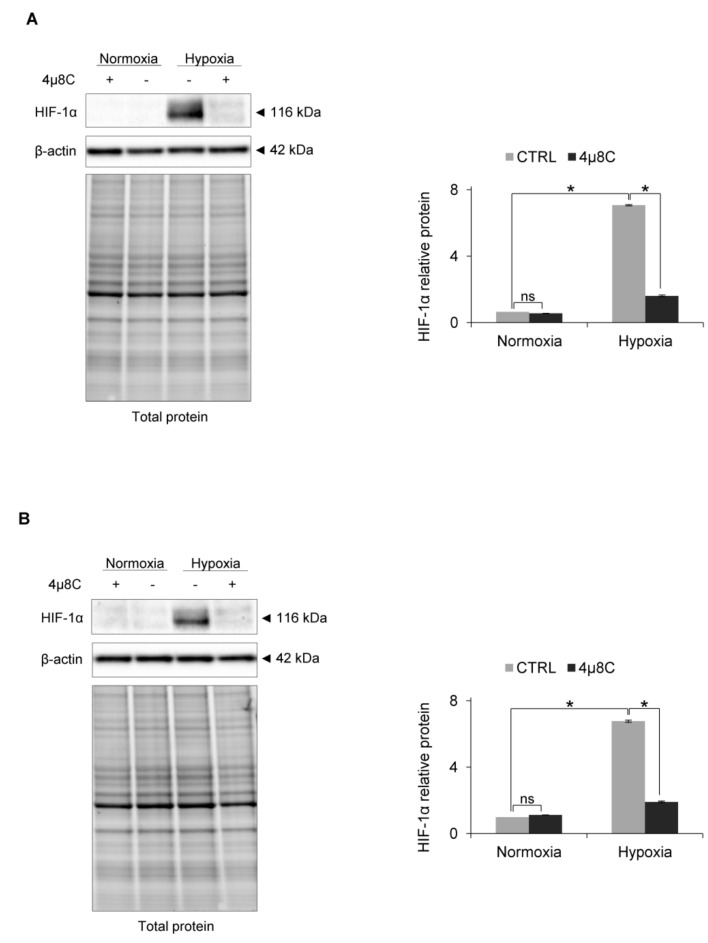
IRE1α inhibition by 4µ8C in hypoxia (6 h) results in HIF-1α protein reduction in (**A**) HMVECs-C and (**B**) HAECs. HIF-1α protein levels after IRE1α inhibition by 4µ8C in normoxia and hypoxia were evaluated by Western Blotting, normalized to β-actin and total protein levels and related to hypoxia (CTRL). The densitometry analysis is representing two independent experiments (* *p* < 0.05 was considered significant).

Finally, we also confirmed the role of IRE1 RIDD activity in HIF-1α accumulation during hypoxia in other primary endothelial human cell lines, including human cardiac microvascular endothelial cells (HMVEC-C) and human aortic endothelial cells (HAEC), where inhibiting IRE1 activity resulted in the dramatic reduction of hypoxic HIF-1α protein (Figure 6A,B).

Taken together, our data show that although the exposure of primary endothelial cells to acute hypoxia does not activate typical UPR signaling during the early time points, the RIDD activity of IRE1 potentiates hypoxic HIF-1α protein accumulation in an XBP1s-independent manner.

## 4. Discussion

Despite the development of novel therapeutic approaches against human cardiovascular and cancer diseases, effective interventions will require determining the mechanisms that regulate cell fate decisions during cellular stress conditions. The problem, however, is that the molecular crosstalk between these pathways remains largely unexplained and limited to cancer cell models [51,52,53,54,55,56,57]. Notably, despite the fact that hypoxia has been reported to activate UPR signaling in order to modulate cancer progression [5,54,58,59], the cancer cell-based models often rely on unique genetic and epigenetic modifications that allow these cells to bypass cell fate decisions during both hypoxia and the UPR. Consequently, deciphering the universal molecular connection between these adaptive responses is very challenging. However, the parallel studies in normal endothelial cells that undergo angiogenesis provide the main adaptive response to an unmet oxygen demand and these types of studies remain underappreciated.

In our approach, we utilized hypoxia-exposed primary HUVECs from 10 pooled donors to determine the role of the ER stress on the HIF-1 signaling pathway. Despite the fact that previous studies reported the hypoxia-related induction of BIP expression [7,60,61,62], we found that *BiP* mRNA levels were reduced after a 12 h exposure to hypoxia. Furthermore, the significant accumulation of apoptotic *CHOP* (*DDIT3)* mRNA was observed only in cells exposed to hypoxia for 24 h. Although CHOP accumulation and the potential induction of an apoptotic response were observed in some hypoxia experiments (including lung endothelial cells) [63,64], these protein and mRNA levels were much lower than those observed during ER stress [19]. Furthermore, we did not observe the accumulation of *XBP1s* that could have suggested the UPR-related activation of IRE1 signaling.

Although we did not test for PERK activation, PERK-mediated eIF2 phosphorylation that could lead to global translational repression was observed in HUVECs within minutes after exposure to acute hypoxia (below 0.1% O_2_), whereas this reaction rate continuously declined with increasing oxygen concentrations [19]. Furthermore, PERK-mediated eIF2 phosphorylation was totally deactivated after 16 h of hypoxia [19]. In our model, we used 0.9% O_2_ and prolonged exposure to hypoxia, and therefore PERK involvement is unlikely. Taken together, our data clearly demonstrate that in HUVECs exposed to hypoxia for up to 12 h, there is no activation of a classical adaptive or apoptotic UPR that could potentially modulate the HIF-1 signals.

Notably, we observed that the hypoxia exposure resulted in early and significantly lower levels of IRE1-processed *XBP1s* mRNA that did not correlate with any significant reduction in the total *XBP1* mRNA expression at 6 h and 12 h. To date, although the accumulation of XBP1s was reported in cancer cell lines exposed to acute and moderate hypoxia [19,20,21,22,23,24,25,26,27], the impairment of *XBP1* splicing under acute hypoxia was also reported [28]. Notably, support for our results came from the study in human pulmonary artery smooth muscle cells (PASMCs) that demonstrated that despite IRE1-related activity, there was no hypoxia-induced XBP1s protein accumulation [29].

Our follow up analysis of IRE1′s role during hypoxia revealed that silencing of this gene paradoxically attenuated HIF-1α accumulation, without apparently affecting *HIF1A* mRNA levels. This observation was further verified by the specific inhibition of IRE1 endoribonuclease activity and supported our view that IRE1′s RIDD activity plays an important role in HIF-1α protein accumulation during hypoxia. Importantly, consistent with the observed hypoxic decline in *XBP1s* mRNA levels, the silencing of *XBP1s* expression during hypoxia has no effect on HIF-1α protein nor mRNA. Although hypoxia did not result in the activation of the classical UPR signaling pathway and *XBP1* expression induction, it is plausible that these transcription factor effects on hypoxic signaling in normal endothelial cells are marginal. Taken together, the data indicate that IRE1 endoribonuclease activity is potentiating hypoxia accumulation of HIF-1α utilizing posttranscriptional pathways that are independent of XBP1s. Furthermore, although hypoxia-related changes in redox balance could affect IRE1 activity, previous studies have shown that oxidative stress attenuates UPR signaling by preventing IRE1 endoribonuclease activity [65]. Therefore, although we cannot exclude ROS-related modifications of IRE1 in our model, these modifications are most likely not related to the observed changes in HIF-1α levels.

Furthermore, it needs to be noted that most ER stress and UPR studies are based on the use of high concentrations of pharmacological ER stressors that result in the potent activation of all UPR pathways at both the transcriptional and posttranscriptional levels [2,41,66]. This is in contrast to the biological role and the extent of the biological activity of PERK and IRE1 in low stress conditions which are relatively unknown. Therefore, we cannot exclude the possibility that although it was not reflected in UPR marker mRNA level measurements, our hypoxia induction over the 12 h period and the related HIF-1 signaling disturbed ER homeostasis, and this led to BIP dissociation from ER membrane and activation of some of the IRE1 and PERK activity, although at much more modest levels than during pharmacological ER stress.

Taken together, although the results presented here provide an important and novel link for IRE1 endoribonuclease activity and HIF-1 signaling during hypoxia in human endothelial cells, the related molecular mechanisms governing IRE1 endoribonuclease hypoxic activity will require further study. We can speculate that during hypoxia, IRE1 activity may support HIF-1α accumulation by degrading specific microRNAs or other RNAs that could lead to HIF-1α translational inhibition in a similar manner as IRE1 mediates the decay of anti-apoptotic microRNAs during the UPR [67]. However, further extensive studies are required to understand IRE1’s role in normal and hypoxic conditions before this hypothesis can be validated.

Finally, we also confirmed the role of IRE1 activity in HIF-1α accumulation during hypoxia in other primary human endothelial cells lines including human cardiac microvascular endothelial cells (HMVEC-C) and human aortic endothelial cells (HAEC), and showed that impairment of RIDD resulted in a dramatic reduction of HIF-1α protein in these cells as well. 

## 5. Conclusions

In summary, we demonstrated that IRE1 endoribonuclease activity is necessary for efficient HIF-1α accumulation in hypoxia-exposed human endothelial cells. However, further studies will be necessary to define the mechanism underlying the molecular relationship between these factors and how they modulate HIF-1-dependent adaptation to low oxygen pressure. The studies presented here have identified IRE1 as a novel player involved in hypoxic HIF-1 signaling.
